# The dangers of parathyroid biopsy

**DOI:** 10.1186/s40463-016-0178-7

**Published:** 2017-01-07

**Authors:** Joanne Kim, Gilad Horowitz, Michael Hong, Mario Orsini, Sylvia L. Asa, Kevin Higgins

**Affiliations:** 1 Department of Otolaryngology (ENT), Sunnybrook Health Sciences Centre, 2075 Bayview Avenue, Toronto, ON M4N 3M5 Canada; 2 Department of Pathology, University Health Network (UHN), 200 Elizabeth Street, 11th Floor, Toronto, ON M5G 2C4 Canada

**Keywords:** Parathyroid, FNA biopsy, Pathology, Diagnostic complication

## Abstract

**Background:**

We report an unusual case of a 66-year-old female with a suspicious thoracic outlet mass presenting with severe biochemical hyperparathyroidism and classic hypercalcemic symptoms of renal and bone involvement.

**Case Presentation:**

There was clinical suspicion for parathyroid carcinoma, further supported by intra-operative findings. However, the final pathology described a primary hyperceullar parathyroid lesion with pathognomonic changes secondary to fine-needle aspiration (FNA) biopsy, along with a separate parathyroid lesion likely resulting from seeding along the needle tract. Upon further review, record of a remote FNA was discovered. This case highlights the complications associated with parathyroid FNA resulting in a diagnostic challenge and raising the possibility of malignancy.

**Conclusions:**

We therefore recommend to take caution when there is a prior parathyroid FNA, as it can present with the risks of a secondary lesion from seeding and increase resemblance of malignancy both clinically and through pathologic diagnosis.

## Background

Ultrasound-guided fine needle aspiration (FNA) can be performed to localize a parathyroid lesion and distinguish parathyroid tissue from surrounding structures [1]. However, there are several innate risks of this procedure that can cause difficulties in diagnosis and management. Specifically, parathyroid adenomas with previous FNA have the risk of a fibrotic reaction leading to adhesions to surrounding structures and histological alterations resembling malignancy [2, 3]. Another potential risk of FNA biopsy is tumor seeding along the needle tract, as parathyroid tissue can adhere to and grow in various settings, explaining the high success with autotransplantations [4–6]. Due to these complications and the limited utility of FNA in typical cases of parathyroid lesions, we would like to encourage caution on interpreting clinical and pathologic diagnoses for cases with a prior FNA.

We report a patient with a six-year history of a slowly enlarging cystic neck mass initially thought to be a benign lymphangioma involving the thoracic outlet. She developed characteristics of parathyroid carcinoma: a palpable neck mass, severe hyperparathyroidism and hypercalcemic symptoms of renal and bone involvement. The lesion was surgically excised and confirmed to be a parathyroid adenoma with extensive fibrosis and cystic degeneration. There was also a separate parathyroid lesion likely resulting from seeding along the needle tract. On careful record review, a report of a previous radiology-directed FNA at an external institution was identified that the patient was unable to recall. Furthermore, there was periprocedural hemolysis and severe ecchymosis that required close observation in the radiology suite. This case emphasizes the complications of fibrosis and seeding secondary to parathyroid FNA.

## Case Presentation and Conclusion

**Fig. 1:**
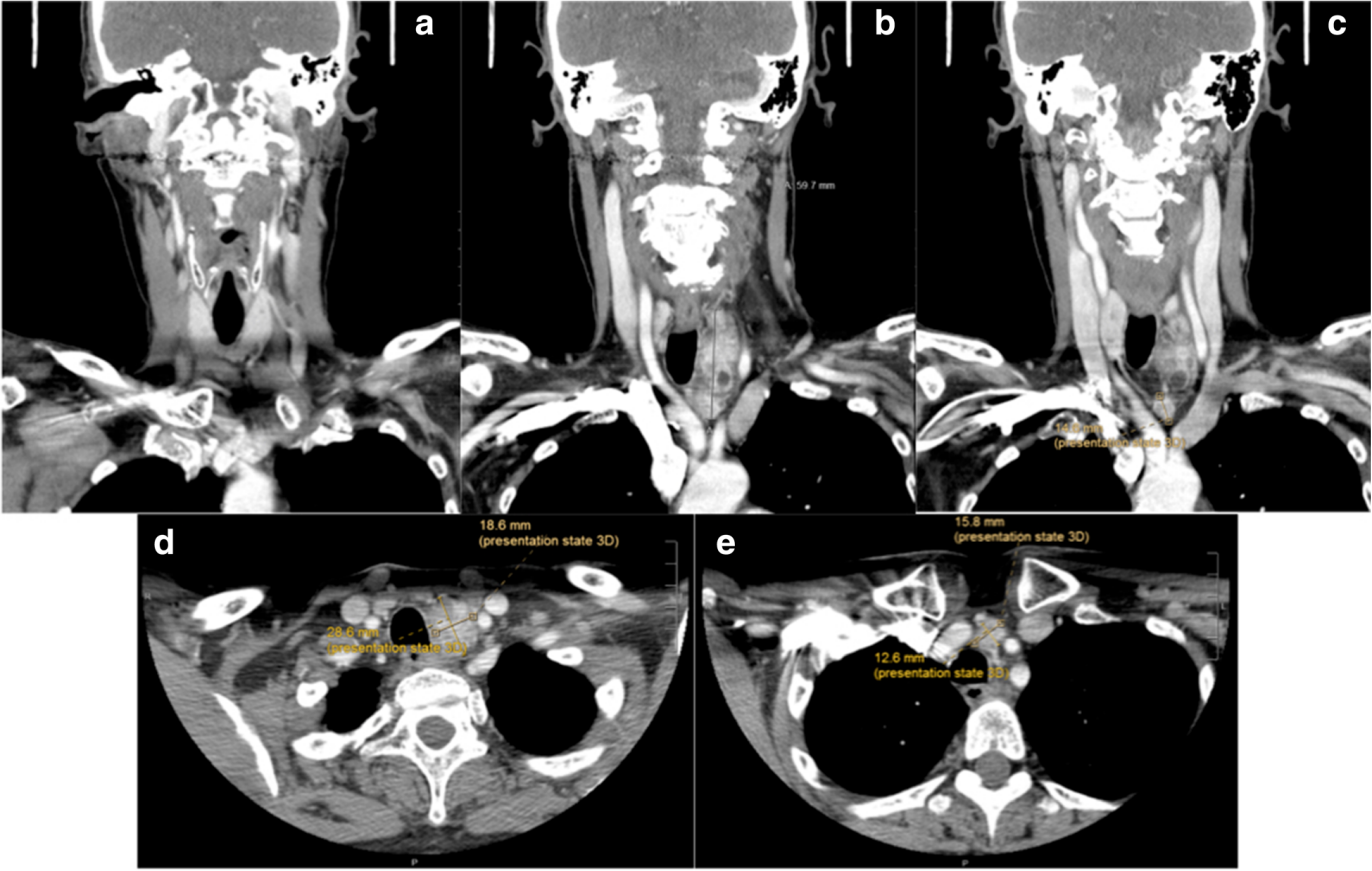
Computed Tomography scan of the neck with IV contrast showing the primary parathyroid lesion and the separate supra-aortic parathyroid lesion, likely secondary to seeding from FNA. **a**, **b**, **c** - Coronal view; **d**, **e** - Axial view. The normal thyroid gland is identified (**a**). The primary parathyroid lesion, measuring 59.7 mm, is located posterior to the thyroid gland (**b**). The separate supra-aortic lesion likely secondary to seeding measures to be 14.6 mm (**c**). The primary parathyroid lesion measures 18.6 mm x 28.6 mm (**d**). The separate supra-aortic lesion localized in the pre-tracheal area measures 12.6 mm x 15.8 mm (**e**)

An otherwise healthy 66-year-old female presented to her family physician with two episodes of hypercalcemia and associated symptoms of fatigue, confusion, visual changes and constipation. Her previous medical history was positive for nephrolithiasis and a long-standing cystic neck mass. This was suspected to be a benign lymphangioma involving the left thoracic outlet, and was followed conservatively by thoracic surgery since 2010. Her bone mass density decreased by over 17% from 2012 to 2014. She was sent to the emergency room where she had a corrected serum calcium (Ca) of 4.54 (2.2–2.7) mmol/L and parathyroid hormone (PTH) of 125 (1.2–5.8) pmol/L. She was admitted and stabilized with IV pamidronate and fluids. She underwent a sestamibi scan with single-photon emission computed tomography (SPECT) showing increased activity in the left cystic neck mass. Repeat investigations showed a corrected Ca of 3.83 (2.2–2.7) mmol/L and normal thyroid function tests. Further investigations included a computed tomography (CT) with IV contrast of the neck and chest showing an enhancing solid and cystic lesion posterior to the left thyroid with extension to the anterior and superior mediastinum of 3.0 × 2.1 × 6.0 cm (AP × TR × CC), juxtaposed between the innominate artery and left common carotid artery down to the aortic arch (Fig. 1). There was no evidence of metastatic disease to the head and neck.

She underwent a left hemithyroidectomy, parathyroidectomy, and modified radical neck dissection of levels III, IV, VI, and VII. The trachea was deviated to the right. Several lymph nodes appeared suspicious prompting the neck dissection. The mass adhered to the surrounding structures and was carefully delineated from the esophagus, trachea and recurrent laryngeal nerve requiring microsurgical dissection. Intra-operative pathology consultation identified hypercellular parathyroid tissue. A pretracheal mass in level VII thought to be nodal was also resected. There were no complications. She presented with a strong voice and cough post-operatively with normal vocal cord movement on fiberoptic nasopharyngoscopy. Post-operative PTH had normalized at 4.3 (1.2–5.8) pmol/L and the corrected Ca also normalized.

**Fig. 2:**
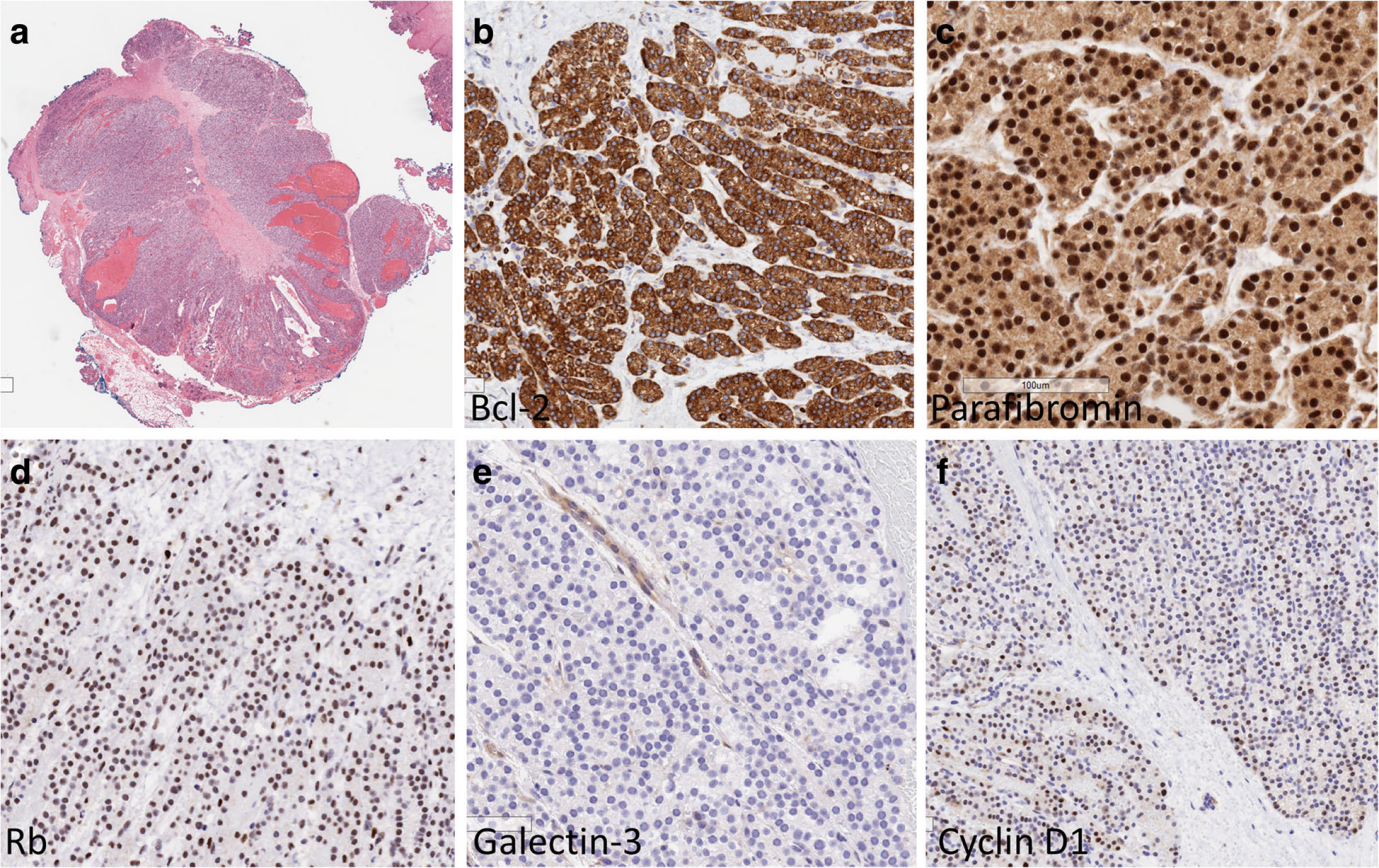
Pathology from the primary parathyroid lesion with FNA-related changes, and biomarker testing to confirm the absence of malignant features. The parathyroid is well delineated but has a central scar; there is hemorrhage and focal cystic change (top left). The tumor is intensely positive for Bcl-2 (top middle). Nuclear parafibromin is intact (top right). Staining identifies Rb in tumor cell nuclei (bottom left). Galectin-3 is not seen in tumor cells; endothelial cells provide an internal positive control (bottom middle). Cyclin D1 is expressed by the majority of the tumor cell nuclei (bottom right)

The final pathology showed two separate lesions composed of similar parathyroid chief and clear cells. Both lesions had extensive fibrosis radiating outwards, and were filled with necrotic debris; there was associated vascularity with hemorrhage and hemosiderin deposition. The larger lesion had surrounding non-tumorous parathyroid tissue that was normocellular. Both specimens had the immunoprofile of non-malignant parathyroid tissue [7]: they stained for GATA-3 and parathyroid hormone, and had intact reactivity for parafibromin, BCL-2, p27 and RB; there was no expression of galectin-3. Cyclin D1 was expressed in the majority of tumor cells, a feature characteristic of parathyroid adenomas; staining for p53 revealed only focal positivity. Ki-67 labelled only about 2% of tumor cells. The thyroid was unremarkable and all lymph nodes were negative for malignancy. The features were consistent with a chief and clear cell adenoma with post-biopsy cystic degeneration and extensive reactive changes (Fig. 2).

There are inherent risks of parathyroid pre-operative biopsies that may alter a typical presentation. Specifically, FNA of parathyroid lesions has the potential for complicating diagnosis and management [1, 8]. Some of the risks include disruption of the lesion and seeding along the needle tract, causing separate secondary parathyroid lesions [4–6]. The dense fibrotic reaction that may occur following FNA can complicate surgery by increasing adhesions to surrounding structures such as the recurrent laryngeal nerve resulting in less clear tumor borders and increased operative time [2]. These features resemble those of parathyroid carcinoma and can also mislead the pathologist with reactive changes atypical for parathyroid adenoma [3].

Due to the rare incidence of parathyroid carcinoma compared to the more commonly seen parathyroid adenoma, clinical suspicion is pertinent to plan appropriate management [9]. Parathyroid carcinoma requires a more extensive treatment plan than parathyroid adenoma, with controversy regarding adjuvant radiation therapy. As the presentation of both lesions are similar and parathyroid carcinoma is very rare, this poses a diagnostic challenge. Pre-operatively, parathyroid carcinoma can be expected based on factors including a larger tumor size, palpable mass, severe primary hyperparathyroidism (PTH 3–15 times the normal upper limit) and hypercalcemia (>3.5 mmol/L) often with renal and bone involvement by the onset of presentation [9]. However, with reactive changes secondary to FNA testing and risk of seeding along the needle tract, these clinical and biochemical differences between a benign and malignant presentation can be further masked making an even more challenging diagnosis. This was illustrated in our case with a large neck mass associated with hyperparathyroidism resembling levels suspicious of malignancy, and hypercalcemic symptoms involving the kidneys and bone.

FNA is known to cause reactive changes seen on histology in the thyroid, salivary gland, breast tissue and parathyroid. These reactive changes include fibrosis and hemosiderin deposition in the chronic setting, and hemorrhage in the acute setting. These features can make it difficult to distinguish between benign and malignant tissue, and therefore histologically will often present with a possibility of malignancy [2, 3]. The use of biomarkers can help to make this distinction and were used for our patient to confirm the absence of proliferative and other proteomic features of malignacy [7]. In our case, specific post-biopsy changes were noted in two tissues. It was unexpected that the second separate lesion would also represent a parathyroid adenoma; we interpret this as a product of seeding from the prior FNA with concomitant histologic post-biopsy changes. This finding is very interesting as it was not suspected pre-operatively on imaging, where the focus was on the prominent larger mass. Hence, when a patient presents with a history of FNA, one must be cautious when the pathologic diagnosis includes a possibility of malignancy as well as being aware of additional discrete lesions that may be secondary to seeding.

Our patient represents a very interesting and unusual case of a parathyroid adenoma with two separate lesions presenting biochemical, clinical and intra-operative features resembling malignancy. The surprising benign outcome on histologic examination confirming a parathyroid adenoma with post-biopsy changes prompted this paper to further expose the negative complications of FNA on parathyroid lesions. As the FNA was completed prior to hospital admission, we were required to search for a record of this investigation to explain the final pathology results. We therefore discourage FNA of parathyroid lesions and if a prior FNA was completed, we encourage caution in the clinical assessment and pathologic interpretation as there is an increased risk of a false-positive for malignancy. In cases presenting with a prior FNA, awareness of the possible fibrotic reaction, histologic alterations and changes in biochemical presentation should be noted when planning patient care.

## Abbreviations

FNA: Fine needle aspiration
PTH: Parathyroid hormone
SPECT: Single-photon emission computed tomography
